# Ticagrelor Versus Clopidogrel in Patients With ST-Elevation Myocardial Infarction and Elevated Platelet Counts: A Multicenter Comparative Analysis of Ischemic Outcomes

**DOI:** 10.31083/RCM46358

**Published:** 2026-05-21

**Authors:** Fang-Jie Ji, Xian Shao, Tian-Shu Gu, Yu-Kun Zhang, Su-Tao Hu, Chao Jiang, Jing-Kun Zhang, Xue Wu, Seung-Woon Rha, Xing Liu, Tong Liu, Kang-Yin Chen

**Affiliations:** ^1^Department of Cardiology, Second Hospital of Tianjin Medical University, Tianjin Key Laboratory of Ionic-Molecular Function of Cardiovascular disease, Tianjin Institute of Cardiology, 300211 Tianjin, China; ^2^Department of Cardiology, The Fourth Central Hospital Affiliated to Tianjin Medical University, 300140 Tianjin, China; ^3^Division of Nephrology, National Clinical Research Center for Kidney Disease, State Key Laboratory of Organ Failure Research, Nanfang Hospital, Southern Medical University, 510515 Guangzhou, Guangdong, China; ^4^Cardiovascular Research Institute, University of California, San Francisco, CA 94158, USA; ^5^Institute for Global Health Sciences, University of California, San Francisco, CA 94158, USA; ^6^Cardiovascular Center, Korea University Guro Hospital, 08308 Seoul, Republic of Korea

**Keywords:** ST-segment elevation myocardial infarction, thrombocytosis, clopidogrel, ticagrelor, prognosis

## Abstract

**Background:**

Dual antiplatelet therapy is essential for managing ST-elevation myocardial infarction (STEMI); however, the optimal choice of P2Y12 inhibitor in patients with thrombocytosis remains unclear. Therefore, this study aimed to compare the effects of clopidogrel and ticagrelor on the prognosis of patients with STEMI and platelet counts exceeding 350 × 10^9^/L.

**Methods:**

Utilizing data from the Tianjin Health and Medical Big Data platform (2010–2023), this retrospective cohort study included patients with acute myocardial infarction from 82 hospitals. After propensity score matching, 461 patients were assigned to two groups: ticagrelor and clopidogrel. Kaplan–Meier curves and Cox regression analyses were employed to evaluate outcomes, with major adverse cardiac and cerebrovascular events (MACCEs) as the primary outcome. Secondary outcomes included net adverse clinical events (NACEs), all-cause mortality, cardiac mortality, recurrent non-fatal myocardial infarction, coronary revascularization, cerebral infarction, and bleeding events (Bleeding Academic Research Consortium (BARC) types 3–5). A MACCE was defined as a composite of cardiac mortality, recurrent non-fatal myocardial infarction, and cerebral infarction, while a NACE encompassed a MACCE plus bleeding events (BARC types 3–5).

**Results:**

Ticagrelor significantly reduced MACCEs (6.9% versus 12.1%; *p* = 0.008), all-cause mortality (3.9% versus 9.5%; *p* < 0.001), cardiac mortality (3.5% versus 7.4%; *p* = 0.0096), and NACEs (8.2% versus 13.0%; *p* = 0.021) compared with clopidogrel. Exploratory multivariable analysis confirmed an independent association of ticagrelor with reduced risks of MACCEs (adjusted hazard ratio (aHR) = 0.59; 95% confidence interval (CI), 0.37–0.93), NACEs (aHR = 0.64; 95% CI, 0.42–0.98), and all-cause mortality (aHR = 0.47; 95% CI, 0.26–0.83).

**Conclusions:**

Ticagrelor was associated with superior clinical outcomes in patients with STEMI and elevated admission platelet counts (≥350 × 10^9^/L) compared with clopidogrel. In contrast to genetic testing, which is costly, time-consuming (≥24–72 hours), and impractical in emergencies, this simple, universally available platelet count threshold offers an immediate, practical biomarker for selecting potent P2Y12 inhibition in acute settings.

## 1. Introduction

Acute ST-segment elevation myocardial infarction (STEMI) constitutes a significant cardiovascular emergency, characterized by high incidence and mortality rates [1, 2]. Current management strategies prioritize dual antiplatelet therapy, with P2Y12 inhibitors serving as critical agents alongside aspirin. Among these, ticagrelor has gained prominence as a preferred option in general STEMI populations owing to its rapid onset and potent, predictable inhibition of platelet aggregation, circumventing the genetic limitations associated with clopidogrel activation via CYP2C19 enzymes. Robust clinical evidence from numerous registries and trials has established the superiority of ticagrelor in mitigating major adverse cardiac and cerebrovascular events (MACCEs) in the general STEMI population [3, 4, 5, 6, 7, 8, 9]. However, the pathophysiological necessity for intensive antiplatelet therapy is considerably heightened in the substantial subset of patients with STEMI presenting with elevated platelet counts. Increasing evidence suggests that higher baseline platelet levels correlate with enhanced platelet reactivity, accelerated thrombus formation, and consequently, poorer clinical outcomes [10, 11, 12, 13]. This hyperreactive platelet phenotype exacerbates atherosclerotic thrombosis and markedly increases the risk of stent thrombosis and recurrent myocardial infarction following percutaneous coronary intervention (PCI), complications that are directly associated with elevated cardiac mortality [14, 15, 16]. Notably, registry data from the Thrombolysis in Myocardial Infarction (TIMI) trials indicate a 34% higher 30-day mortality in patients with STEMI and platelet counts exceeding the median value compared with that in patients with lower counts [10]. In East Asian populations, large-scale studies further delineate specific high-risk thresholds, such as admission platelet counts ≥350 × 10^9^/L, which are linked to heightened 30-day mortality (hazard ratio (HR) = 1.34; 95% confidence interval (CI), 1.12–1.61) [10] and a 28% increased risk of recurrent ischemic events [17]. These findings underscore the urgent need for optimized antiplatelet strategies in this high-risk subgroup exhibiting a hypercoagulable state.

Despite this compelling pathophysiological rationale and consistent clinical observations, current guidelines lack specific recommendations for selecting platelet therapy based on platelet count. Moreover, existing comparative studies on P2Y12 inhibitors predominantly focus on broad STEMI populations, potentially obscuring differential treatment effects that may be crucial for patients with a heightened thrombotic propensity attributed to thrombocytosis [3, 4, 5, 6]. This knowledge gap is clinically significant, considering that over 40% of patients with STEMI present with platelet counts exceeding normal ranges at admission [10]. This phenomenon yields a skewed distribution (median: 218 × 10^9^/L, interquartile range (IQR): 178–268 × 10^9^/L) where values ≥350 × 10^9^/L—beyond the upper quartile (Q3 of approximately 268 × 10^9^/L)—represent a well-defined, extremely high-risk subset comprising approximately 5.8% of cases in large cohorts (for example, 8742/150,530 patients with acute myocardial infarction (AMI)), characterized by amplified platelet reactivity and poor prognosis [10, 17]. Coupled with unacceptably high stent thrombosis rates (2.9%–5.8%) even under contemporary therapies [15], these data underline the importance of targeting antiplatelet optimization toward this vulnerable group.

To directly address this critical therapeutic void, our study performed the first head-to-head comparison of ticagrelor with clopidogrel, exclusively in patients with STEMI presenting with admission thrombocytosis. By elucidating the optimal antiplatelet regimen for this vulnerable population, we aimed to (1) provide evidence-based guidance specifically designed to alleviate their excessive thrombotic risk attributed to elevated platelet counts and (2) enhance long-term cardiovascular outcomes. This study adopted an elevated platelet count (≥350 × 10^9^/L) as an inclusion criterion, with the threshold selection supported by robust clinical and epidemiological evidence. In East Asian populations, large-scale studies have identified admission platelet counts ≥350 × 10^9^/L as a clinically meaningful high-risk threshold. The original observation of graded mortality risk with higher platelet counts came from the TIMI trials [10], while subsequent large Korean registries, including the Korea Acute Myocardial Infarction Registry-National Institutes of Health (KAMIR-NIH), confirmed that the risk increases sharply and becomes statistically significant precisely at ≥350 × 10^9^/L, with an adjusted HR of 1.34 (95% CI, 1.12–1.61) for 30-day mortality and an approximately 28% higher risk of recurrent ischemic events in East Asian patients with STEMI [10, 17]. This cutoff effectively enriches for patients in a hypercoagulable state, aligning with prior evidence and facilitating the detection of differential antiplatelet treatment effects.

## 2. Methods

### 2.1 Study Design and Population

The data for this study were derived from the coronary artery disease (CAD) specialized database within the Tianjin Health and Medical Big Data Super Platform (referred to as the “platform”). Tianjin Health and Medical Big Data Co., Ltd. serves as the authorized data provider responsible for the collection, management, and application of data on this platform. The platform aggregates clinical diagnosis and treatment information from 43 tertiary and 39 secondary hospitals in the Tianjin region, as well as data from the public health system.

After normalization and de-identification on the platform, the data were transformed into a specialized database for scientific research on the CAD database, which includes patients who were hospitalized at least once between January 1, 2010, and June 30, 2023, with discharge diagnoses of CAD. Comprehensive healthcare information for these patients was collected, encompassing demographic characteristics, disease diagnoses, medication and non-medication prescriptions, examination and laboratory test results, surgical information, cost details, community medication and health examination data, as well as public health mortality information.

**Fig. 1.**
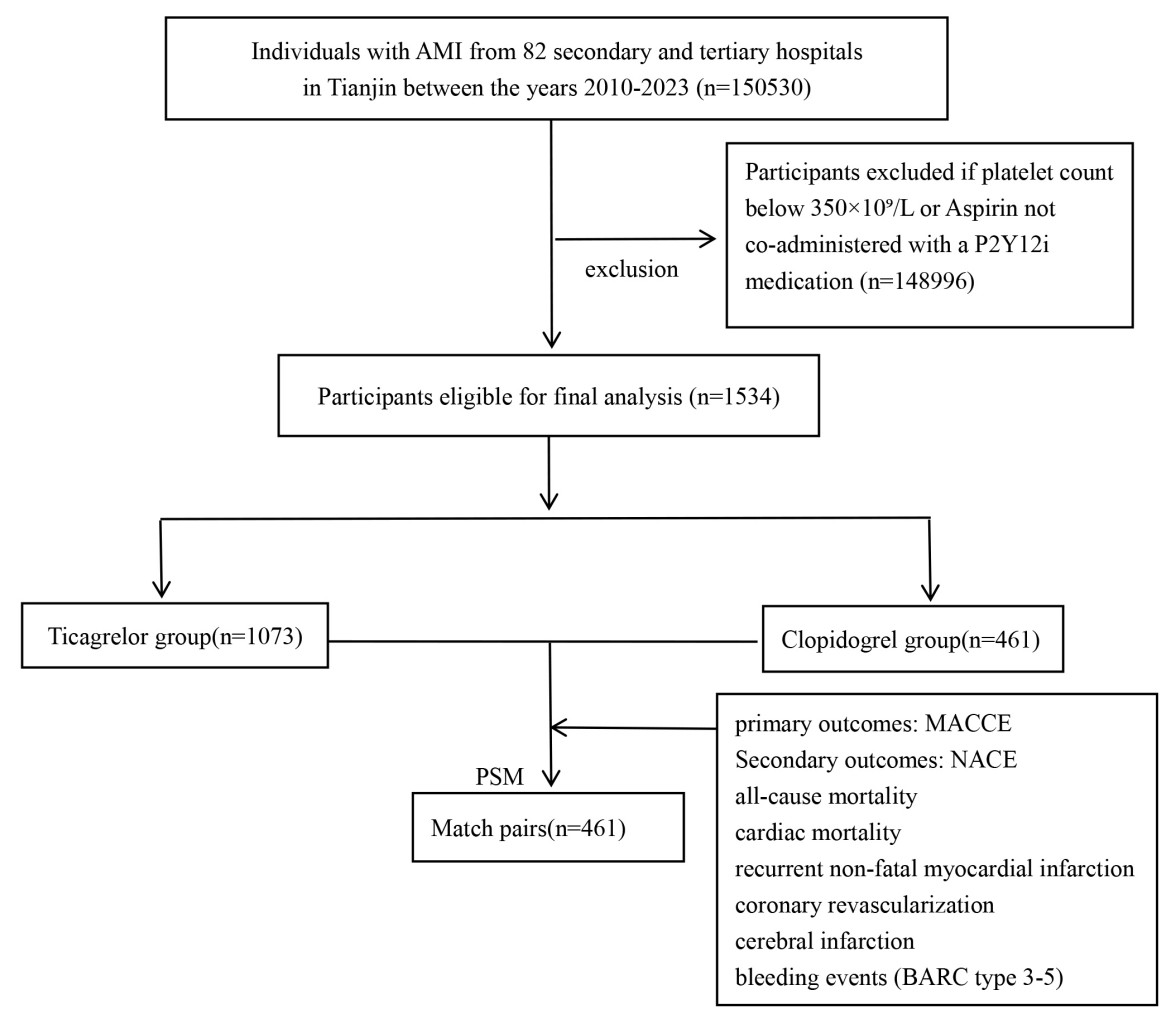
**Study flowchart**. MACCE, major adverse cardiac and cerebrovascular event; NACE, net adverse clinical event; AMI, acute myocardial infarction; PSM, propensity score matching; BARC, Bleeding Academic Research Consortium.

This study employed the International Classification of Diseases, 10th Revision (ICD-10) diagnostic codes to identify patients discharged with a diagnosis of STEMI. Following STEMI diagnosis, patients were prescribed aspirin as a baseline medication, in addition to either ticagrelor or clopidogrel. Patients were required to have an initial admission platelet count ≥350 × 10^9^/L, a threshold established by large-scale East Asian registry studies, including the TIMI trials [10] and the Korea Acute Myocardial Infarction Registry-National Institutes of Health [17], both of which demonstrated that platelet counts at or above this level are independently associated with significantly increased short- and long-term adverse outcomes in patients with STEMI [10, 17]. This cutoff corresponds to the uppermost range (>Q3 + 1.5 × IQR) in the platelet count distribution of contemporary East Asian AMI cohorts, thereby enriching for a subgroup with markedly heightened platelet reactivity and thrombotic risk. ICD-10 codes were utilized to extract data on complications and comorbidities, including ventricular fibrillation, ventricular tachycardia, third-degree atrioventricular block, diabetes, hypertension, and atrial fibrillation (Table 1). The overall flowchart is illustrated in Fig. 1. Among 150,530 patients with AMI, 1534 were included in the study. These patients were categorized into two groups based on their use of ticagrelor (n = 1073) or clopidogrel (n = 461) during hospitalization. Propensity score matching (PSM) at a 1:1 ratio was performed between the two groups, resulting in 461 patients being analyzed for 1-year follow-up survival. The extracted database population solely comprised patients on aspirin who were treated exclusively with either ticagrelor or clopidogrel, and all patients maintained consistent antiplatelet therapy without any in-hospital changes. Dual antiplatelet therapy was initiated at the discretion of the physician and in accordance with the European Society of Cardiology (ESC) guidelines [18]. In non-PCI patients, tertiary centers frequently preferred ticagrelor for its intensified antithrombotic effect, particularly in cases of thrombocytosis. This study was conducted in accordance with the guiding principles of the Declaration of Helsinki and received approval from the Clinical Research Ethics Committee of the Second Hospital of Tianjin Medical University (KY2023052-01), which waived the requirement for informed patient consent.

**Table 1.** **Baseline clinical characteristics**.

Characteristics		Before PSM				After PSM			
		Clopidogrel group	Ticagrelor group	*p* value	SMD	Clopidogrel group	Ticagrelor group	*p* value	SMD
		N = 1073	N = 461			N = 461	N = 461		
Male (%)		608 (56.7)	297 (64.4)	0.005	0.159	298 (64.6)	297 (64.4)	1.000	0.005
Age (%)		62.0 (14.08)	56.3 (13.06)	<0.001	0.417	56.0 (13.56)	56.0 (13.06)	0.970	0.002
Killip class (%)				<0.001				0.872	
	I	590 (55.0)	297 (64.4)			307 (66.6)	297 (64.4)		
	II	274 (25.5)	122 (26.5)			111 (24.1)	122 (26.5)		
	III	119 (11.1)	18 (3.9)			18 (3.9)	18 (3.9)		
	IV	90 (8.4)	24 (5.2)			25 (5.4)	24 (5.2)		
Complications									
	Ventricular fibrillation (%)	19 (1.8)	9 (2.0)	0.972	0.013	9 (2.0)	9 (2.0)	1.000	<0.001
	Ventricular tachycardia (%)	43 (4.0)	11 (2.4)	0.153	0.092	19 (4.1)	11 (2.4)	0.194	0.098
	III atrioventricular block (%)	12 (1.1)	5 (1.1)	1.000	0.003	4 (0.9)	5 (1.1)	1.000	0.022
Comorbidities									
	Diabetes mellitus (%)	289 (26.9)	116 (25.2)	0.510	0.040	116 (25.2)	116 (25.2)	1.000	<0.001
	Hypertension (%)	387 (36.1)	169 (36.7)	0.870	0.012	168 (36.4)	169 (36.7)	1.000	0.005
	Atrial fibrillation (%)	19 (1.8)	6 (1.3)	0.656	0.038	5 (1.1)	6 (1.3)	1.000	0.020
	Hyperlipidemia (%)	383 (35.7)	186 (40.3)	0.095	0.096	182 (39.5)	186 (40.3)	0.840	0.018
	Chronic obstructive pulmonary disease (%)	25 (2.3)	8 (1.7)	0.586	0.042	7 (1.5)	8 (1.7)	1.000	0.017
	Stroke (%)	194 (18.1)	74 (16.1)	0.376	0.054	79 (17.1)	74 (16.1)	0.723	0.029
	Cerebral hemorrhage (%)	105 (9.8)	27 (5.9)	0.016	0.147	33 (7.2)	27 (5.9)	0.504	0.153
	Renal insufficiency (%)	167 (15.6)	73 (15.8)	0.954	0.007	62 (13.4)	73 (15.8)	0.352	0.068
	Peripheral vascular disease (%)	92 (8.6)	41 (8.9)	0.916	0.011	34 (7.4)	41 (8.9)	0.470	0.056
	Previous PCI (%)	16 (1.5)	2 (0.4)	0.132	0.108	5 (1.1)	2 (0.4)	0.448	0.175

Footnotes: Data are presented as n (%) and the mean (standard deviation (SD)) for categorical and continuous (age) variables, respectively. *p* values were calculated using the χ^2^ or Fisher’s exact test for categorical variables and Student’s *t*-test for age. Standardized mean differences (SMDs) were reported pre- and post-matching; a post-matching SMD <0.1 indicated balance. PSM was performed using a 1:1 nearest-neighbor matching algorithm without replacement, with a caliper width of 0.2 of the SD of the propensity score logit. Matching covariates included age, sex, Killip class, and all listed comorbidities and complications. After matching, the SMDs for all covariates were less than 0.1, suggesting adequate balance. PCI, percutaneous coronary intervention; PSM, propensity score matching.

### 2.2 Data Collection and Treatment

This study recorded basic information and clinical data, including sex, age, Killip grade, and comorbidities (hypertension, hyperlipidemia, diabetes, atrial fibrillation, stroke, cerebral hemorrhage, renal insufficiency, and peripheral vascular disease). Hospitalization and discharge medications were also documented, including aspirin, oral anticoagulants, statins, nitrates, beta-blockers, renin–angiotensin system inhibitors (RASIs; angiotensin-converting enzyme inhibitors (ACEIs), angiotensin II receptor blockers (ARBs), or angiotensin receptor-neprilysin inhibitors (ARNIs)), calcium channel antagonists, diuretics, levosimendan, and amiodarone. Laboratory tests included D-dimer and fibrinogen assays. Data collection was conducted by trained physicians using post-discharge electronic health records. Patients with AMI were treated according to established guidelines—unless contraindicated or explicitly rejected by family members—with dual antiplatelet therapy, coronary artery reperfusion therapy, and subsequent ventricular anti-remodeling. Successful PCI was defined as achieving post-procedure TIMI grade 3 flow with <30% residual stenosis. PCI success rates were comparable between groups after matching (96.8% versus 95.7%, *p* = 0.480).

### 2.3 Definition of Outcomes

In this study, MACCE constituted the primary outcome, while secondary outcomes included net adverse clinical events (NACEs), all-cause mortality, cardiac mortality, recurrent non-fatal myocardial infarction, coronary revascularization, cerebral infarction, and bleeding events (Bleeding Academic Research Consortium (BARC) type 3–5). MACCE were specifically defined as cardiac mortality, recurrent non-fatal myocardial infarction, and cerebral infarction, while NACE encompassed cardiac mortality, recurrent non-fatal myocardial infarction, cerebral infarction, and bleeding events (BARC type 3–5).

The study outcomes included both in-hospital adverse events and 1-year adverse outcomes. Primary outcome measures comprised in-hospital and 1-year cardiac mortalities. Secondary in-hospital adverse outcomes included malignant arrhythmias not excluded from the study cohort, specifically ventricular tachycardia, ventricular fibrillation, and ventricular flutter. Secondary 1-year adverse outcomes included all-cause mortality, follow-up myocardial infarction, revascularization events, stroke events, and bleeding events classified as BARC type 3–5. Follow-up myocardial infarctions were defined as subsequent hospital admissions for AMI recorded in the database after the baseline event. Stroke was defined as new hospital admissions within 1 year owing to either new cerebral infarction or hemorrhage. Revascularization events were defined as any revascularization procedure performed on any segment of the coronary artery, including branch vessels, after discharge from the baseline AMI. “BARC type 3–5” events were defined as bleeding incidents that satisfy the BARC criteria for types 3 to 5, as recorded in the database. The breakdown is as follows: type 3a (overt bleeding with a hemoglobin decline of 3–5 g/dL or transfusion of 1–2 units), type 3b (hemoglobin decline ≥5 g/dL, transfusion ≥3 units, or intervention), type 3c (intracranial bleeding), type 4 (coronary artery bypass grafting-related major bleeding), and type 5 (fatal: 5a probable, 5b confirmed). Incidence values are presented in Table 1. Follow-up was censored at 12 months or the last known vital status.

### 2.4 Statistical Analysis

We conducted a complete-case analysis, ensuring no missing data or imputation of missing values. All statistical analyses were performed using R software (version 4.3.2; R Foundation for Statistical Computing, Vienna, Austria). Variable selection for the exploratory multivariable Cox proportional hazards regression model employed a combined clinical and statistical approach. Clinically relevant covariates identified in prior literature (for example, age, sex, Killip class, hypertension, diabetes mellitus, prior stroke, renal insufficiency, and in-hospital PCI) were included in the model irrespective of their univariate significance. Additional variables with a *p* value < 0.10 in univariate analyses were also considered for inclusion. All statistical tests were two-sided, with statistical significance set at *p* < 0.05.

This analysis adhered to a predefined statistical analysis plan established before data extraction from the Tianjin Health and Medical Big Data Platform. The study protocol and analytic framework received approval from the Clinical Research Ethics Committee of the Second Hospital of Tianjin Medical University (KY2023052-01). Although the study was not prospectively registered in a public trial registry, all primary and secondary endpoints, covariate definitions, and model specifications were prespecified before statistical analysis commenced.

PSM was performed using a 1:1 nearest-neighbor matching algorithm without replacement, with caliper width set to 0.2 times the SD of the propensity score logit. 1:1 propensity score matching was performed using a greedy nearest-neighbor algorithm, maintaining a 1:1 ratio and a caliper width of 0.02 times the SD of the propensity score logit. Matched variables included sex, age, Killip classification, receipt of PCI during hospitalization, prior history of diabetes, hypertension, stroke, or hyperlipidemia, and the use of calcium channel blockers, beta-blockers, RASIs, statins, nitrates, or oral anticoagulants. Following the matching process, inter-group balance was evaluated using SMDs, with values less than 0.1 indicating adequate balance.

We compared baseline characteristics between the ticagrelor and clopidogrel groups pre- and post-PSM. Continuous variables are presented as the mean ± SD or median IQR, and they were compared using independent-sample *t*-tests or the Wilcoxon rank-sum test, as appropriate. Categorical variables are reported as frequencies (percentages), and they were compared using the Chi-square or Fisher’s exact test.

To achieve doubly robust estimation, inverse probability treatment weighting (IPTW) was applied to the full cohort, incorporating reperfusion variables (primary PCI (PPCI), elective PCI). Sensitivity analyses included Fine–Gray competing risk models for non-fatal endpoints (with death as a competing event) and landmark Cox models for timing-specific outcomes (in-hospital, 0–30 days, and 31–365 days). Subgroup analyses, based on age, sex, PPCI, renal function, and GPI use, employed interaction tests (*p* for interaction).

Survival analyses utilized Kaplan–Meier curves to estimate the cumulative incidence of adverse events, with the log-rank test employed to compare survival differences between treatment groups. Exploratory multivariable Cox proportional hazards regression was applied to evaluate the independent association between ticagrelor use and study outcomes. Additionally, due to the limited number of events (62 all-cause deaths and 50 cardiac deaths), the full multivariable Cox models that adjusted for nine prespecified covariates yielded a low events-per-variable ratio (approximately 6–7). These multivariable-adjusted hazard ratios should therefore be regarded as exploratory and interpreted with caution. The primary evidence for the benefits of ticagrelor derives from the more robust propensity score-matched intention-to-treat comparison and doubly robust IPTW analyses.

## 3. Results

### 3.1 Study Population Characteristics 

As depicted in Tables 1,2, significant pre-PSM differences were noted between the clopidogrel and ticagrelor groups. The ticagrelor group exhibited a greater proportion of men (64.4% versus 56.7%, *p* = 0.005) and a lower mean age (56.3 versus 62.0 years, *p* < 0.001). The distribution of Killip classes also differed significantly (*p* < 0.001), with more patients falling under Killip Class I in the ticagrelor group. Additionally, the ticagrelor group displayed higher rates of PCI (74.4% versus 54.5%, *p* < 0.001) and PPCI (69.6% versus 44.4%, *p* < 0.001). Certain complications and comorbidities, such as ventricular fibrillation, diabetes, and hypertension, demonstrated no significant pre-PSM differences between the two groups.

**Table 2.** **In-hospital treatments**.

	Before PSM				After PSM			
	Clopidogrel group	Ticagrelor group	*p* value	SMD	Clopidogrel group	Ticagrelor group	*p* value	SMD
	N = 1073	N = 461			N = 461	N = 461		
PCI (%)	585 (54.5)	343 (74.4)	<0.001	0.425	348 (75.5)	343 (74.4)	0.761	0.025
PPCI (%)	476 (44.4)	321 (69.6)	<0.001	0.528	291 (63.1)	321 (69.6)	0.043	0.138
Thrombolytic (%)	22 (2.1)	11 (2.5)	0.823	0.023	9 (2.0)	11 (2.4)	0.821	0.030
Aspirin (%)	1073 (100.0)	461 (100.0)	1.000	< 0.001	461 (100.0)	461 (100.0)	1.000	<0.001
Oral anticoagulant (%)	17 (1.6)	1 (0.2)	0.043	0.145	0 (0.0)	1 (0.2)	1.000	0.066
RASIs (%)	641 (59.7)	339 (73.5)	<0.001	0.296	334 (72.5)	339 (73.5)	0.767	0.024
β-Blockers (%)	785 (73.5)	371 (80.5)	0.003	0.174	370 (80.3)	371 (80.5)	1.000	0.005
Calcium channel blockers (%)	100 (9.3)	40 (8.7)	0.761	0.002	41 (8.9)	40 (8.7)	1.000	0.008
Nitrates (%)	571 (53.2)	156 (33.8)	<0.001	0.398	160 (34.7)	156 (33.8)	0.835	0.018
Diuretic (%)	517 (48.2)	159 (34.5)	<0.001	0.281	178 (38.6)	159 (34.5)	0.218	0.086
Statins (%)	1043 (97.2)	460 (99.8)	0.002	0.213	461 (100.0)	460 (99.8)	1.000	0.066
Levosimendan (%)	11 (1.0)	5 (1.1)	1.000	0.006	2 (0.4)	5 (1.1)	0.448	0.075
Amiodarone (%)	31 (2.9)	8 (1.7)	0.255	0.077	9 (2.0)	8 (1.7)	1.000	0.016
Fib, g/L	4.06 ± 1.48	3.77 ± 1.20	<0.001	0.363	3.85 ± 1.37	3.77 ± 1.20	0.363	0.060
D-D, mg/L	1.02 ± 1.68	0.78 ± 1.33	0.006	0.745	0.81 ± 1.05	0.78 ± 1.33	0.745	0.021

Footnotes: Data are presented as n (%) and the mean ± SD for categorical and continuous (Fib and D-D) variables, respectively. *p* values were calculated using χ^2^ or Fisher’s exact tests for categorical variables and independent-samples Student’s *t*-tests for continuous variables. PSM utilized the same 1:1 nearest-neighbor matching algorithm as in Table 1 (caliper width = 0.2 of the SD of the propensity score logit, without replacement). In-hospital treatments were not included in the propensity score model; post-PSM differences reflect residual confounding or treatment selection bias not entirely adjusted for baseline characteristics. PCI, percutaneous coronary intervention; PPCI, primary percutaneous coronary intervention; PSM, propensity score matching; RASIs, renin–angiotensin system inhibitors (including ACEIs and ARBs); Fib, fibrinogen; D-D, D-Dimer.

Post-PSM, the differences were largely mitigated, enhancing comparability between the groups. The proportions of men and the mean ages were balanced (*p* = 1.000 and *p* = 0.970, respectively), and the distribution of Killip classes was similar (*p* = 0.872). PCI usage varied insignificantly (*p* = 0.761), although the PPCI rate remained slightly higher in the ticagrelor group (69.6% versus 63.1%, *p* = 0.043), representing potential residual confounding. Medication usage, including RASIs, β-blockers, nitrates, and diuretics, exhibited no significant post-PSM differences. Laboratory parameters, such as fibrinogen and D-dimer levels, were also comparable between the groups. This matching procedure established a more robust basis for comparing clinical outcomes between the two treatment groups. The C-statistic for the logistic regression model used in propensity score estimation was 0.709, indicating acceptable discriminative ability.

### 3.2 Clinical Outcomes 

**Fig. 2.**
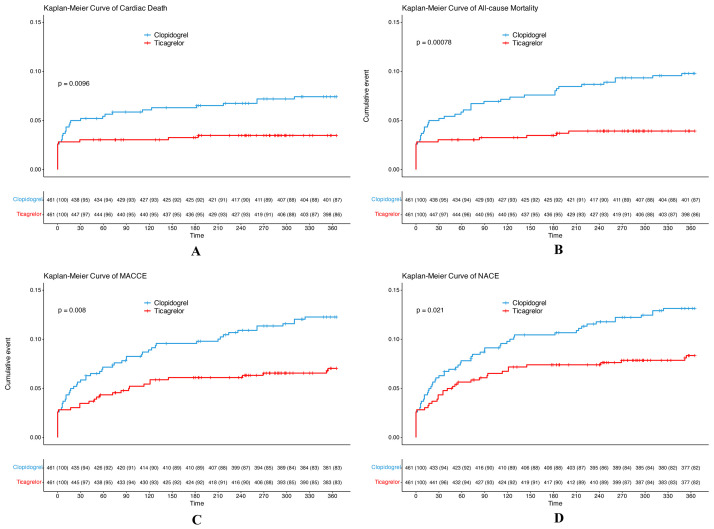
**Kaplan–Meier survival curves illustrating cumulative incidences of various 1-year clinical outcomes in propensity score-matched patients ((A) Cardiac death; (B) All-cause death; (C) MACCE; and (D) NACE)**. Footnotes: The curves depict 1 minus the Kaplan–Meier survival probability, representing the cumulative incidence function. The step functions are constructed based on event times within specified intervals, with risk tables displaying at-risk numbers and censoring counts positioned below the x-axis at designated time points (days: 0, 30, 60, 90, 120, 150, 180, 210, 240, 270, 300, and 360). The y-axes are standardized to a 0%–25% scale. *p* values were obtained through a log-rank test comparing clopidogrel to ticagrelor within a propensity score-matched cohort (n = 461 per group at baseline). The analysis employed an intention-to-treat approach, with follow-up censored at 360 days or upon event/death. Abbreviations: MACCE, major adverse cardiac and cerebrovascular event (cardiac death/non-fatal myocardial infarction/cerebral infarction); NACE, net adverse clinical event (MACCE plus BARC type 3–5 bleeding).

The clinical outcomes of the study, as detailed in Table 3 and Fig. 2, demonstrate significant differences in the effectiveness of ticagrelor compared with that of clopidogrel in patients with STEMI presenting with elevated platelet counts.

**Table 3.** **Association of primary and secondary outcomes with ticagrelor versus clopidogrel in propensity score-matched patients**.

		Clopidogrel group	Ticagrelor group	*p* value
		N = 461	N = 461	
Primary outcome				
	MACCE (%)	56 (12.1)	32 (6.9)	0.008
Secondary outcomes				
	NACE (%)	60 (13.0)	38 (8.2)	0.021
	All-cause Mortality (%)	44 (9.5)	18 (3.9)	<0.001
	Cardiac Deaths (%)	34 (7.4)	16 (3.5)	0.0096
	Recurrent MI (%)	15 (3.3)	11 (2.4)	0.40
	Revascularization (%)	16 (3.5)	19 (4.1)	0.64
	New Stroke (%)	9 (2.0)	6 (1.3)	0.41
	BARC type 3–5 (%)	4 (0.9)	6 (1.3)	0.52

Footnotes: The primary (MACCE: cardiac mortality, recurrent non-fatal myocardial infarction, and cerebral infarction) and secondary (NACE: MACCE plus BARC type 3–5 bleeding events) endpoints are presented. Statistical significance was set at *p* < 0.05. Cox HRs with 95% CIs from IPTW-adjusted models are included. Follow-up was censored at 360 days or upon event/death. Type 3–5 bleeding is defined according to the BARC criteria, as detailed in the Methods section. IPTW, inverse probability treatment weighting; MACCE, major adverse cardiac and cerebrovascular event; NACE, net adverse clinical event; BARC, Bleeding Academic Research Consortium.

The primary outcome, MACCE, was significantly less pronounced in the ticagrelor group (6.9%) than in the clopidogrel group (12.1%, *p* = 0.008). Similarly, NACE rates were significantly lower in the ticagrelor group (8.2%) than in the clopidogrel group (13.0%, *p* = 0.021). Furthermore, the ticagrelor group demonstrated a substantial reduction in all-cause mortality (3.9%) relative to the clopidogrel group (9.5%, *p* < 0.001). Cardiac deaths occurred less frequently in the ticagrelor group (3.5%) than in the clopidogrel group (7.4%, *p* = 0.0096).

Nonetheless, other secondary outcomes—recurrent myocardial infarction, revascularization, new stroke, and “BARC type 3–5” bleeding events—revealed no significant differences between the two groups. Recurrent myocardial infarction incidence rates were 3.3% and 2.4% in the clopidogrel and ticagrelor groups, respectively (*p* = 0.40). The revascularization rate was 3.5% in the clopidogrel group and 4.1% in the ticagrelor group (*p* = 0.64). New strokes occurred in 2.0% and 1.3% of the clopidogrel and ticagrelor groups, respectively (*p* = 0.41). “BARC type 3–5” bleeding events were reported at 0.9% in the clopidogrel group and 1.3% in the ticagrelor group (*p* = 0.52). BARC type 3 or higher bleeding events refer to those classified by the BARC as types 3 to 5, as documented in the database.

**Fig. 3.**
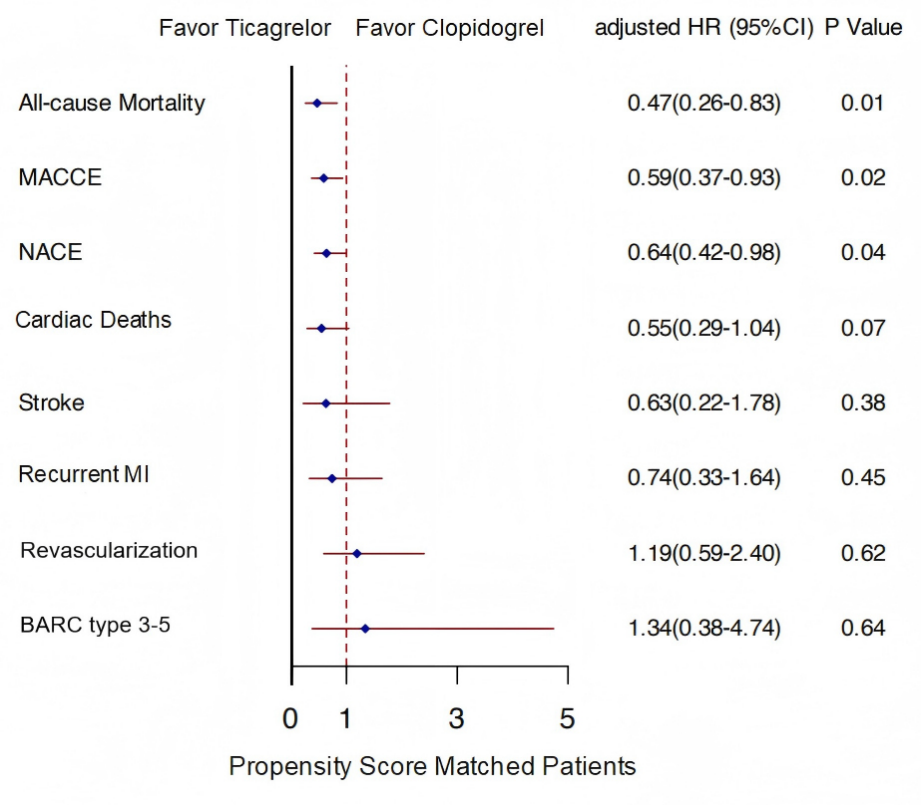
**Adjusted forest plot of clinical outcomes with median follow-up time**. Footnotes: The forest plot displays exploratory multivariable-adjusted HRs with 95% CIs derived from Cox proportional hazards regression models within the propensity score-matched cohort (N = 461 per group). The vertical line at HR = 1.0 indicates no difference between groups; points to the left of 1.0 favor ticagrelor, whereas those to the right favor clopidogrel. Models were adjusted for all baseline characteristics listed in Table 1 (including age, sex, Killip class, comorbidities, and complications), as well as in-hospital treatments that were significantly imbalanced after matching (primary PCI). The proportional hazards assumption was verified using Schoenfeld residuals (global *p* > 0.05 for all outcomes). Follow-up was censored at 12 months or at the last known vital status. MACCE: cardiac mortality, recurrent non-fatal myocardial infarction, and cerebral infarction; NACE: MACCE plus BARC type 3–5 bleeding. BARC type 3–5 refers to major bleeding events classified by the BARC.

Fig. 3 corroborates these conclusions by presenting aHRs and 95% CIs. The aHR for MACCE was 0.59 (95% CI, 0.37–0.93; *p* = 0.02), indicating a significantly reduced risk with ticagrelor. Regarding NACE, the aHR was 0.64 (95% CI, 0.42–0.98; *p* = 0.04). All-cause mortality yielded an aHR of 0.47 (95% CI, 0.26–0.83; *p* = 0.01), demonstrating a significant survival benefit associated with ticagrelor. Nevertheless, no statistically significant differences were noted for cardiac deaths, recurrent MI, revascularization, new stroke, or “BARC type 3–5” bleeding events (*p* > 0.05).

IPTW was applied to the entire pre-matching cohort for doubly robust estimation. After IPTW, baseline characteristics were well balanced (all SMD <0.14; Table 4). IPTW-adjusted Cox proportional hazards models confirmed the robustness of the main findings, demonstrating a significant reduction in 1-year MACCE (HR 0.605, 95% CI, 0.378–0.969, *p* = 0.036) and consistent trends toward lower all-cause mortality (HR 0.557, 95% CI, 0.299–1.036, *p* = 0.064) and NACE (HR 0.654, 95% CI, 0.425–1.008, *p* = 0.054) with ticagrelor (Table 5). Fine–Gray competing-risk models accounting for the competing risk of death produced similar results for non-fatal endpoints (**Supplementary Table 1**).

**Table 4.** **Baseline characteristics comparison after IPTW weighting (Ticagrelor versus Clopidogrel)**.

Characteristic		Clopidogrel (weighted n = 1543.2)	Ticagrelor (weighted n = 1473.9)	*p* value	SMD
Demographic					
	Male (%)	913.5 (59.2%)	903.1 (61.3%)	0.506	0.042
	Age, years (mean ± SD)	60.03 ± 14.38	59.13 ± 12.91	0.283	0.066
Killip class (%)				0.914	0.048
	I	897.2 (58.1%)	887.4 (60.2%)		
	II	396.0 (25.7%)	368.9 (25.0%)		
	III	135.5 (8.8%)	120.7 (8.2%)		
	IV	114.4 (7.4%)	96.9 (6.6%)		
Complications					
	Ventricular fibrillation (%)	29.7 (1.9%)	21.5 (1.5%)	0.505	0.036
	Ventricular tachycardia (%)	64.1 (4.2%)	36.3 (2.5%)	0.170	0.095
	Third-degree atrioventricular block (%)	15.2 (1.0%)	16.3 (1.1%)	0.861	0.012
Comorbidities					
	Diabetes mellitus (%)	582.8 (37.8%)	543.5 (36.9%)	0.776	0.018
	Hypertension (%)	556.8 (36.1%)	567.9 (38.5%)	0.442	0.051
	Atrial fibrillation (%)	198.4 (12.9%)	142.4 (9.7%)	0.118	0.101
	Hyperlipidemia (%)	571.9 (37.1%)	545.0 (37.0%)	0.978	0.002
	Chronic obstructive pulmonary disease (%)	34.3 (2.2%)	24.5 (1.7%)	0.492	0.041
	Stroke (%)	266.3 (17.3%)	237.0 (16.1%)	0.623	0.032
	Cerebral hemorrhage (%)	53.2 (3.4%)	48.4 (3.3%)	0.879	0.009
	Renal insufficiency (%)	230.7 (14.9%)	297.0 (20.1%)	0.053	0.137
	Previous PCI (%)	24.7 (1.6%)	4.6 (0.3%)	0.015	0.133

IPTW, inverse probability treatment weighting; PCI, percutaneous coronary intervention.

**Table 5.** **Hazard ratios from IPTW-adjusted Cox models (in-hospital ticagrelor versus clopidogrel, 1-year outcomes)**.

Outcome	HR	95% CI lower	95% CI upper	*p* value
All-cause death	0.557	0.299	1.036	0.064
MACCE	0.605	0.378	0.969	0.036
NACE	0.654	0.425	1.008	0.054
Myocardial infarction	0.779	0.366	1.657	0.516
Revascularization	1.977	1.035	3.776	0.039
New-onset stroke	0.392	0.155	0.991	0.048
BARC 3 bleeding	0.862	0.340	2.187	0.755
Cardiovascular death	0.668	0.347	1.288	0.229

IPTW, inverse probability treatment weighting; MACCE, major adverse cardiac and cerebrovascular event; NACE, net adverse clinical event; BARC, Bleeding Academic Research Consortium.

### 3.3 Sensitivity Analysis by Treatment Era

Sensitivity analyses stratified by treatment era (2010–2018 versus 2019–2023) showed consistent benefit of ticagrelor in the more recent period (2019–2023), with significant reductions in 1-year all-cause death (HR 0.412, 95% CI, 0.182–0.934), MACCE (HR 0.451, 95% CI, 0.244–0.834), and NACE (HR 0.503, 95% CI, 0.278–0.908), whereas no significant differences were observed in the earlier period (Table 6).

**Table 6.** **Stratified analyses by period (2010–2018 versus 2019–2023)**.

Period	Outcome	HR	95% CI	*p* value
2010–2018	DEATH_1Y	0.507	(0.185–1.388)	0.187
	MACCE_1Y	0.735	(0.338–1.597)	0.436
	NACE_1Y	0.819	(0.408–1.645)	0.574
	MI_1Y	1.909	(0.427–8.532)	0.397
	Revascularization_1Y	0.983	(0.205–4.722)	0.983
	NGS_1Y	0.541	(0.144–2.023)	0.361
	BARC3-5_1Y	3.086	(0.141–67.623)	0.474
	CD_1Y	0.863	(0.308–2.420)	0.780
2019–2023	DEATH_1Y	0.412	(0.182–0.934)	0.034
	MACCE_1Y	0.451	(0.244–0.834)	0.011
	NACE_1Y	0.503	(0.278–0.908)	0.023
	MI_1Y	0.362	(0.134–0.983)	0.046
	Revascularization_1Y	1.086	(0.456–2.583)	0.852
	NGS_1Y	1.780	(0.154–20.581)	0.644
	BARC3-5_1Y	1.965	(0.192–20.156)	0.570
	CD_1Y	0.405	(0.165–0.996)	0.049

MACCE, major adverse cardiac and cerebrovascular event; NACE, net adverse clinical event; BARC, Bleeding Academic Research Consortium; MI, myocardial infarction; NGS, new-onset stroke; CD, cardiac death.

### 3.4 Subgroup Analyses

Pre-specified subgroup analyses (age, sex, primary PCI, glycoprotein IIb/IIIa inhibitor (GPI) use) showed no significant heterogeneity of treatment effect for most outcomes, although numerical trends favoured greater benefit of ticagrelor in patients without primary PCI and without GPI use (all *p*_interaction > 0.05 except marginal trends; **Supplementary Table 2**).

### 3.5 Landmark Analysis

Landmark analysis showed that the mortality benefit of ticagrelor was particularly pronounced beyond the first 30 days, with a significant reduction in all-cause death from day 31 to 365 (0.9% versus 4.6%; *p* = 0.001). The reduction in all-cause death within the first 30 days did not reach statistical significance (3.0% versus 5.0%; *p* = 0.179). For cardiovascular death, no statistically significant difference was noted in the first 30 days (3.0% versus 5.0%; *p* = 0.179), while a significant reduction was identified during the 31–365 day period (0.4% versus 2.4%; *p* = 0.025) (Table 7).

**Table 7.** **Timing-specific mortality rates and hazard ratios (IPTW-adjusted, ticagrelor versus clopidogrel)**.

Time period	Outcome	Clopidogrel (n = 461)	Ticagrelor (n = 461)	*p* value	SMD
0–30 days	All-cause death	23 (5.0%)	14 (3.0%)	0.179	0.100
	Cardiovascular death	23 (5.0%)	14 (3.0%)	0.179	0.100
31–365 days	All-cause death	21 (4.6%)	4 (0.9%)	0.001	0.229
	Cardiovascular death	11 (2.4%)	2 (0.4%)	0.025	0.166

IPTW, inverse probability treatment weighting.

## 4. Discussion

Utilizing the Tianjin Health and Medical Big Data Platform (2010–2023), this multicenter retrospective cohort study performed the first direct comparison of ticagrelor with clopidogrel in patients with STEMI presenting with admission thrombocytosis (platelet count ≥350 × 10^9^/L). After PSM (n = 461 per group), ticagrelor significantly reduced the primary outcome (MACCE: 6.9% versus 12.1%; *p* = 0.008) and secondary outcomes, including NACE (8.2% versus 13.0%; *p* = 0.021), all-cause mortality (3.9% versus 9.5%; *p* < 0.001), and cardiac mortality (3.5% versus 7.4%; *p* = 0.0096). Multivariate Cox analysis validated ticagrelor as an independent protective factor against MACCE (aHR = 0.59; 95% CI, 0.37–0.93), NACE (aHR = 0.64; 95% CI, 0.42–0.98), and all-cause mortality (aHR = 0.47; 95% CI, 0.26–0.83). No significant differences were detected in bleeding events (BARC type 3–5) or other ischemic endpoints, emphasizing the superior ischemic benefit–risk profile of ticagrelor in this high-thrombotic-risk subgroup.

Elevated platelet counts are a significant risk factor for cardiovascular diseases, particularly myocardial infarction and stroke. When exceeding 350 × 10^9^/L, they increase cardiovascular risk by promoting thrombosis, obstructing vessels, and exacerbating myocardial ischemia in patients with STEMI. Platelets also drive inflammatory responses, correlating with coronary endothelial damage and heightened inflammation. Accordingly, platelet count serves as a key indicator for assessing STEMI prognosis, with studies indicating poorer outcomes and increased cardiovascular events in patients with thrombocytosis [14, 15].

Mechanistically, the advantages of ticagrelor over clopidogrel in this context arise from its more potent and reversible P2Y12 receptor inhibition, circumventing CYP2C19-dependent activation limitations. Furthermore, beyond stronger P2Y12 inhibition, ticagrelor exhibits pleiotropic effects that may be particularly relevant in the proinflammatory and prothrombotic milieu of thrombocytosis. By inhibiting the equilibrative nucleoside transporter 1 (ENT1), ticagrelor increases circulating adenosine, which exerts anti-inflammatory, vasodilatory, and microvascular protective effects. Elevated platelet counts are frequently reactive to systemic inflammation and are associated with endothelial dysfunction and impaired coronary microcirculation. These adenosine-mediated actions may therefore contribute additional benefit in this high-inflammatory-burden subgroup, complementing the primary antiplatelet effect and further explaining the pronounced ischemic and mortality reductions observed. For instance, ticagrelor has demonstrated potential in improving coronary microvascular function in experimental sepsis [19] and has exhibited satisfactory antiplatelet effects at lower doses compared with clopidogrel [20]. Additionally, ticagrelor and its active metabolites effectively inhibit platelet function [21].

These findings build upon extensive prior research evaluating antiplatelet therapy in STEMI, where ticagrelor has consistently outperformed clopidogrel in broader populations. The ESC guidelines advocate for ticagrelor as the first-line treatment option for patients with STEMI undergoing PCI [18]. Furthermore, post-PCI ticagrelor treatment is preferred for patients with the CYP2C19 loss-of-function allele to mitigate MACCE [22]. Prehospital administration of ticagrelor prior to PCI has yielded improved outcomes in patients with STEMI [23, 24, 25]. In a Korean study [17], ticagrelor was found to reduce the risk of MACCE in patients with AMI and multivascular disease compared to clopidogrel. Notably, the superior efficacy of ticagrelor observed in this study aligns with these prior findings. Although the high prevalence of CYP2C19 loss-of-function alleles in East Asian populations provides a plausible biological explanation for this consistent benefit, this study did not genotype patients, leaving the contribution of pharmacogenetic variations to our findings speculative and warranting further investigation. These benefits remained robust after adjustments for calendar time, site, and PPCI. A nationwide cohort study [26] suggests that ticagrelor may be beneficial in preventing post-myocardial infarction stroke in East Asian patients.

Although the lack of CYP2C19 genotyping is a limitation, admission platelet count ≥350 × 10^9^/L offers distinct practical advantages. Genetic testing is costly, requires specialized facilities, and typically delays results by ≥24–72 h—unacceptable in acute STEMI. In contrast, platelet count is routinely available within minutes of admission at virtually no additional cost. This simple, universally accessible biomarker allows immediate identification of high-thrombotic-risk patients who derive the greatest benefit from ticagrelor, enabling rapid, evidence-based P2Y12 inhibitor selection without waiting for pharmacogenetic results.

With regard to safety, observational data comparing ticagrelor with clopidogrel are inconsistent. The PEGASUS–TIMI 54 trial [27] found ticagrelor to significantly reduce the incidence of cardiovascular death, myocardial infarction, and stroke, although it also resulted in an increased risk of major bleeding in patients with a myocardial infarction history extending beyond 1 year. Additionally, transitioning from clopidogrel to ticagrelor significantly improved 1-year clinical outcomes without an increased risk of bleeding [28]. Notwithstanding, most studies have not specifically investigated high platelet counts in patients with STEMI, limiting our understanding of this subgroup. To date, few studies have examined the prognosis of antiplatelet therapy in patients with STEMI and elevated platelet counts.

Regarding safety, the low absolute number of BARC type 3–5 bleeding events (1.3% versus 0.9%, *p* = 0.52) precludes definitive conclusions about bleeding risk and indicates limited statistical power for this low-event endpoint, with a high risk of Type II error (false negative). However, in this particularly high-thrombotic-risk cohort (admission platelet count ≥350 × 10^9^/L), ticagrelor achieved marked reductions in all-cause mortality (aHR 0.47), cardiac mortality, and MACCE without a significant increase in major bleeding. This pattern strongly suggests a favorable net clinical benefit, in which the substantial prevention of fatal and ischemic events clearly outweighs any modest or undetected increase in bleeding hazard, supporting the preferential use of ticagrelor in patients with STEMI and thrombocytosis.

## 5. Strengths and Limitations 

### 5.1 Strengths

This study leveraged a vast dataset from 82 secondary and tertiary hospitals in Tianjin City, covering the 2010–2023 period and encompassing a sizable number of patients with AMI. This extensive sample size enhances statistical power and overall reliability. Furthermore, the analysis assessed multiple primary and secondary clinical outcomes, including MACCE, NACE, all-cause mortality, cardiac mortality, recurrent non-fatal myocardial infarction, coronary artery revascularization, stroke, and bleeding events (BARC type 3–5). Such a comprehensive evaluation enabled an in-depth comparison of the efficacy of ticagrelor against that of clopidogrel in patients with STEMI and thrombocytosis.

The observed benefits of ticagrelor—characterized by a significantly lower rate of cardiovascular events than that associated with clopidogrel, with a more pronounced advantage than in previous trials—are attributable to the high-risk patient population, optimized treatment protocols, and rigorous follow-up strategies. Additionally, the high prevalence of CYP2C19 intermediate metabolizers (approximately 40%–45%) within the Han population [29], driven by loss-of-function allele carrier rates of 38.6% for CYP2C192 and 5.2% for CYP2C193, likely enhanced the superiority of ticagrelor, as it circumvents the metabolic limitations of clopidogrel. Although pharmacogenetic data were not directly incorporated, this ethnic-specific context further strengthens the relevance of the findings for East Asian cohorts.

### 5.2 Limitations 

Despite the significantly lower incidence of cardiovascular events associated with ticagrelor than with clopidogrel—potentially attributable to the high-risk thrombocytosis subgroup, optimized protocols, and rigorous follow-up—several limitations warrant consideration.

First, as an administrative database study, endpoint classifications (for example, cardiac death and all-cause mortality) relied solely on ICD codes without independent adjudication of pathological causes, potentially introducing misclassification bias. Furthermore, the database did not capture detailed causes of cardiovascular death (for example, pump failure, reinfarction-related death, stent thrombosis, arrhythmic death, procedure-related death, or unknown cause), limiting the ability to perform sub-classifications and direct comparisons of incidence rates between groups. This constraint hinders a more comprehensive exploration of the factors driving survival benefits, suggesting that future studies should enhance detailed death adjudication.

Second, several non-fatal secondary endpoints exhibited low absolute event counts (for example, recurrent myocardial infarction: 11 versus 15; BARC type 3–5 bleeding: 6 versus 4), thus limiting the statistical power to detect meaningful differences. Additionally, post-PSM sample sizes (n = 461 per group) were modest, potentially yielding insufficient power for rarer outcomes and increasing the risk of false negatives.

Third, all data were sourced from hospitals in Tianjin, where regional variations in medical standards, treatment practices, and population characteristics limit the generalizability of the findings to broader Chinese or international populations. Caution is thus warranted when extrapolating results on a nationwide or global scale.

Fourth, despite stringent inclusion and exclusion criteria, residual selection bias might have persisted owing to patient heterogeneity, variability in treatment strategies, and inconsistencies in follow-up duration. The Killip classification, derived from discharge documentation, may not accurately reflect admission severity; although adjusted for shock proxies (vasopressors, intubation, and intra-aortic balloon pump/extracorporeal membrane oxygenation), residual misclassification remains possible and requires prospective validation. While temporal adjustments increased robustness, residual secular trends are acknowledged as limitations. Moreover, post-discharge medication adherence was unmeasured, and reactive thrombocytosis was not adjusted for owing to the unavailability of markers—factors warranting future exploration via claims data integration and marker assessment. Despite PSM and IPTW, residual confounding may persist, including the slight post-PSM imbalance in primary PCI rate (*p* = 0.043), which could influence ischemic outcomes favoring ticagrelor. However, the consistency of benefits in IPTW-adjusted models (which included reperfusion variables) and sensitivity analyses mitigates this concern.

Fifth, the database lacks longitudinal outpatient medication dispensing or refill data. As a result, post-discharge adherence to the initially prescribed P2Y12 inhibitor, rates of treatment switching, and discontinuation could not be evaluated. The intention-to-treat analysis assuming persistent exposure to the discharge medication may therefore underestimate or overestimate the true on-treatment effect of ticagrelor versus clopidogrel. Importantly, this limitation most likely introduces a conservative bias that underestimates the true magnitude of ticagrelor’s benefit. In an ITT framework, patients initially prescribed ticagrelor who subsequently discontinued therapy or switched to clopidogrel (e.g., due to dyspnea, cost, or physician preference) would be analyzed in the ticagrelor arm despite receiving reduced or no exposure to the drug. The fact that highly significant reductions in MACCE, all-cause mortality, and cardiac mortality were still observed despite this probable dilution of treatment effect strongly supports a robust biological advantage of ticagrelor in this high-thrombotic-risk population and validates the observed benefits. Future studies incorporating pharmacy claims linkage or prospective follow-up are needed to address adherence and persistence in this high-risk population. Future research should prioritize the assessment of bleeding risks across diverse platelet functional states and implement targeted preventive measures.

Additionally, due to the limited number of events (62 all-cause deaths and 50 cardiac deaths), the full multivariable Cox models that adjusted for nine prespecified covariates yielded a low events-per-variable ratio (approximately 6–7). These analyses are exploratory, and the multivariable-adjusted hazard ratios should therefore be regarded as exploratory and interpreted with caution. The primary evidence for the benefits of ticagrelor derives from the more robust propensity score-matched intention-to-treat comparison and doubly robust IPTW analyses, which are not subject to the same events-per-variable constraint.

Although the subgroup analyses were prespecified and adjusted for multiple comparisons where appropriate, the nominal interactions observed for MACCE (*p*_interaction = 0.039) and NACE (*p*_interaction = 0.047) in patients with versus without primary PCI should be interpreted with considerable caution. These interactions are driven by low event counts in several subgroups and composite endpoints that include non-fatal events of lesser clinical severity, rendering them underpowered for detecting true heterogeneity of treatment effect. Accordingly, no definitive claims of subgroup-specific differences are made, and the overall findings remain consistent across the majority of tested subgroups.

To address these limitations, subsequent studies should adopt prospective designs with comprehensive data collection (for instance, detailed death adjudication, genotype profiling, and precise timing of platelet measurements), minimize biases through multicenter recruitment, and enhance generalizability via nationwide cohorts.

## 6. Conclusions

In patients with STEMI and elevated admission platelet counts (≥350 × 10^9^/L), oral ticagrelor was associated with substantial reductions in all-cause mortality, cardiac mortality, and major adverse cardiovascular events. In contrast to CYP2C19 genetic testing—which is impractical in acute settings due to high cost, need for specialized facilities, and 24–72-hour delays—the admission platelet count is universally available within minutes at no extra cost. This simple biomarker enables rapid, point-of-care identification of high-thrombotic-risk patients who benefit most from ticagrelor, supporting immediate evidence-based antiplatelet selection in this population.

## Availability of Data and Materials

Please contact the corresponding authors for access to the data.
