# Supplementary material for: Ticagrelor Versus Clopidogrel in Patients With ST-Elevation Myocardial Infarction and Elevated Platelet Counts: A Multicenter Comparative Analysis of Ischemic Outcomes
Source: Rev Cardiovasc Med. 2026 May 21;27(5):46358. doi: 10.31083/RCM46358 (PMC13227351; doi:10.31083/RCM46358)
Supplement: Supplementary file 1 [file 2153-8174-27-5-46358-s1.zip › Supplementary Tables.docx]

**Supplementary Table 1: Hazard Ratios from IPTW-Adjusted Cox Models (In-Hospital Ticagrelor vs. Clopidogrel, 1-Year Outcomes)**

| **Nonfatal Endpoint** | **Sub-HR** | **95% CI** | **P Value** |
| --- | --- | --- | --- |
| Ischemic Stroke | 0.231 | (0.110–0.486) | <0.001 |
| Acute MI (AMI) | 0.664 | (0.396–1.110) | 0.12 |
| Revascularization | 0.712 | (0.462–1.110) | 0.12 |
| BARC type 3-5 | 0.752 | (0.242–2.330) | 0.62 |

These analyses reinforce the robustness of our conclusions regarding ticagrelor's safety and efficacy profile.

**Supplementary Table 2: Subgroup Analyses by Key Covariates: Hazard Ratios (HRs) for Ticagrelor vs. Clopidogrel (1-Year Outcomes)**

| **Subgroup** | **All-Cause Death (HR [95% CI]; P_int)** | **CV Death (HR [95% CI]; P_int)** | **MACCE (HR [95% CI]; P_int)** | **NACE (HR [95% CI]; P_int)** |
| --- | --- | --- | --- | --- |
| **Age** |  |  |  |  |
| **<75 years** | 0.40 [0.14–1.13] | 0.52 [0.16–1.73] | 0.77 [0.41–1.44] | 0.85 [0.49–1.50] |
| **≥75 years** | 0.56 [0.28–1.13] | 0.69 [0.32–1.45] | 0.56 [0.29–1.07] | 0.60 [0.32–1.13] |
| **P_interaction** | 0.594 | 0.704 | 0.473 | 0.403 |
| **Sex** |  |  |  |  |
| **Female** | 0.76 [0.38–1.53] | 0.91 [0.41–1.99] | 0.93 [0.49–1.78] | 0.89 [0.48–1.65] |
| **Male** | 0.25 [0.08–0.76] | 0.37 [0.12–1.16] | 0.50 [0.27–0.96] | 0.64 [0.36–1.14] |
| **P_interaction** | 0.097 | 0.203 | 0.187 | 0.460 |
| **PPCI** |  |  |  |  |
| **No** | 0.30 [0.11–0.82] | 0.36 [0.12–1.10] | 0.36 [0.16–0.80] | 0.41 [0.19–0.88] |
| **Yes** | 0.81 [0.39–1.71] | 1.02 [0.45–2.31] | 1.02 [0.57–1.83] | 1.05 [0.62–1.78] |
| **P_interaction** | 0.121 | 0.139 | 0.039 | 0.047 |
| **GPI Use (Tirofiban)** |  |  |  |  |
| **No** | 0.24 [0.10–0.59] | 0.26 [0.09–0.76] | 0.41 [0.21–0.81] | 0.44 [0.23–0.82] |
| **Yes** | 1.31 [0.55–3.11] | 1.47 [0.60–3.60] | 1.10 [0.58–2.08] | 1.26 [0.69–2.29] |
| **P_interaction** | 0.008 | 0.015 | 0.039 | 0.017 |
| **Overall** | 0.53 [0.30–0.94] | 0.67 [0.36–1.26] | 0.68 [0.43–1.07] | 0.75 [0.49–1.14] |

Footnote: IPTW-adjusted Cox proportional hazards models for 1-year outcomes in STEMI patients with elevated platelets (>350×10⁹/L). HRs compare ticagrelor vs. clopidogrel. P_interaction tests heterogeneity between PPCI strata. No significant interactions (all P_int >0.05).
